# Thoracolumbar paravertebral giant ganglioneuroma and scoliosis: a case report and literature review

**DOI:** 10.1186/s12957-016-0823-7

**Published:** 2016-03-05

**Authors:** Yihao Yang, Mingyan Ren, Zhongqin Yuan, Kun Li, Zhiping Zhang, Jing Zhang, Lin Xie, Zuozhang Yang

**Affiliations:** Department of Orthopaedics, The Third Affiliated Hospital of Kunming Medical University, Tumor Hospital of Yunnan Province, Kunming, Yunnan 650118 People’s Republic of China; Department of Medical Oncology, The Third Affiliated Hospital of Kunming Medical University, Tumor Hospital of Yunnan Province, Kunming, Yunnan 650118 People’s Republic of China; Department of Radiology, The Third Affiliated Hospital of Kunming Medical University, Tumor Hospital of Yunnan Province, Kunming, 650118 People’s Republic China

**Keywords:** Ganglioneuroma, Scoliosis, Thoracolumbar spine, Tumor resection

## Abstract

Paravertebral ganglioneuroma and scoliosis is a rare clinical benign disease. The case we reported is about a 12-year-old girl who was hospitalized due to neoplasm with spinal deformity in the right abdomen for 1 month. Based on a careful preoperative evaluation and found no obvious surgery contraindications, the patient was treated with surgical resection of the tumor and correction of the deformity by surgery. Postoperative pathologic examination confirmed it was a ganglioneuroma. After the operation, the patient recovered well. Her spinal deformity was corrected, and she was 5 cm taller. Complete resection of ganglioneuroma following with a low recurrence rate and a good prognosis, patient does not need further chemotherapy, radiation therapy, or other treatments. All follow-up radiographic studies demonstrated no relapse of the tumor in the following 18 months. Combining this case with similar cases at home and aboard and reviewing related literature, we formed conclusions based on the manifestations, diagnosis, treatment, and prognosis of this disease and provided treatments for similar cases.

## Background

Ganglioneuromas are rare benign tumors that originate from a neural crest or sympathetic ganglion [1]. They most commonly appear in the posterior mediastinum and abdomen [2]. The patients exhibit no obvious symptoms upon nervous system examination. The ganglioneuromas are often found in females, while the male/female ratio is approximately 2/3 [3]. The incidence of ganglioneuroma is not well documented, but it is estimated to characterize 0.1 to 0.5 % of total central nervous system (CNS) tumors [4]. Paravertebral ganglioneuroma and scoliosis is rarer and has only been sporadically reported. In this report, we present a case of thoracolumbar paravertebral ganglioneuroma in a 12-year-old girl who presented with scoliosis. The study and analysis of the case improved the knowledge of this tumor, and related literature was incorporated to improve the understanding of the disease.

## Case presentation

**Fig. 1:**
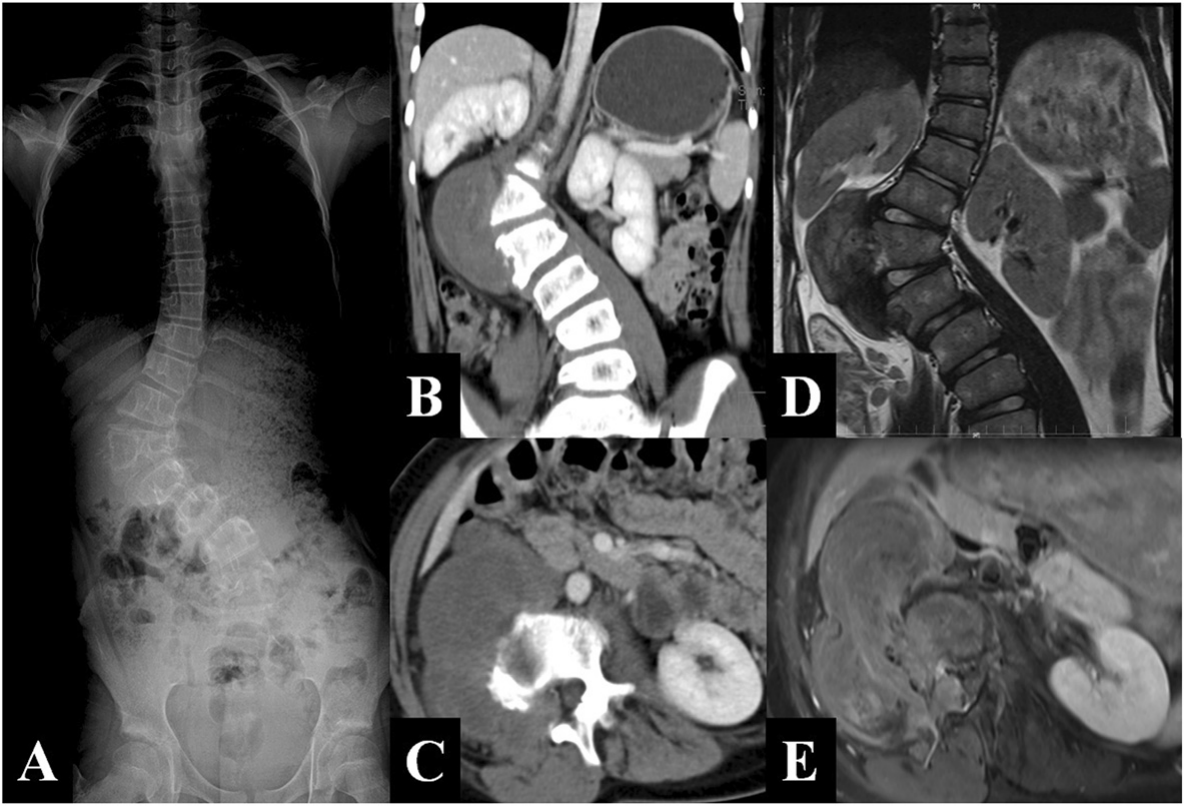
**a** The lower segment of the thoracic and lumbar spine existed a right tumefied thoracolumbar curve surrounding the first lumbar body with a Cobb angle of 33.7° and Ferguson angle of 69.4°. **b**, **c** Axial CT scans and coronary multi-plane reorganization demonstrated a right paravertebral irregular soft tissue mass with low and nonhomogeneous density, as well as uneven strength from the T_12_ to L_2_ vertebrae. The tumor grows through L_1/2_ right intervertebral foramen lesions to spinal canal. The intervertebral foramen becomes larger, and the right side of L_2_ vertebrae with irregular bone becomes corroded damage. **d**, **e** Axial enhanced MRI scan demonstrated the tumor is less homogeneous reinforcement, grows through L_1/2_ right intervertebral foramen lesions to spinal canal. The spinal shift to the left side due to cord compression

A 12-year-old girl visited our hospital with “a right abdominal mass and spinal deformity 1 month” on July 22, 2014. When she was 3, the girl was received a mass resection operation in the local hospital, but without postoperative pathologic examination. She recovered well after the surgery. At the age of 5, her parents found that their daughter had mild claudication, but they did not take it seriously until 1 month ago, when she complained of a right abdominal mass with mild pain. In the meantime, the parents noticed the girl had scoliosis. She was then hospitalized in our orthopedics department. The child did not have a family history of tumors. Upon physical examination, we found an old vertical operation scar sized 5 cm in the right abdominal region. In the right lumbar region, we identified a lump measuring 10 cm × 7 cm that was solid, without clear boundaries, immobilized, and having no tenderness. A lumbar right curvature could be observed, and there was no direct or indirect percussion pain or neurological deficits. From a radiographic examination, the lower segment of the thoracic and lumbar spine showed a right tumefied thoracolumbar curve and left thoracolumbar curve around the 1st lumbar body at a Cobb angle of 33.7° and Ferguson angle of 69.4° (Fig. 1a), respectively. A CT and an MRI showed a paravertebral soft tissue mass from the T_12_ to L_2_ vertebrae (Fig. 1b–e). The preoperative value of NSE was 17.51 μg/L↑. Other laboratory examinations revealed no other abnormalities.

**Fig. 2:**
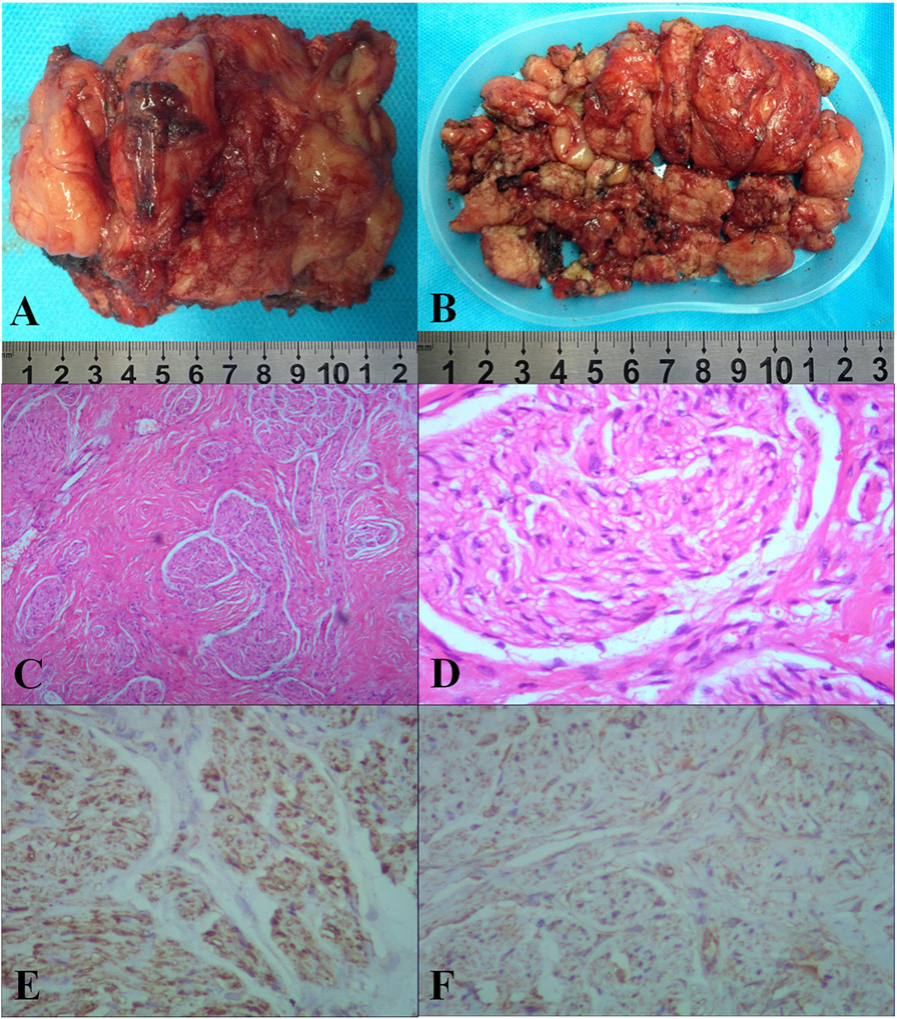
**a** The tumor which was removed in the first operation was 11 cm × 7 cm × 4 cm, well encapsulated, luidity section, solid, and tough texture. **b** The tumor which was removed in the second operation, measuring 13 cm × 8 cm × 6 cm, has the same character with that in the first operation. **c**, **d** 4 × 10, 40 × 10 magnification photomicrograph, respectively, hematoxylin and eosin. The tumor consists of mature ganglion cells, and the oncocyte distributed in mesenchyme with different amounts glial-fibrous tissue. **e** 20 × 10 magnification photomicrograph, immunohistochemistry, S-100(+). **f** 20 × 10 magnification photomicrograph, immunohistochemistry, Vim(+)

The large tumor was adjacent to important organs such as the right kidney and inferior vena cava. Moreover, the patient presented with scoliosis. Therefore, a staging operation was performed. The first stage operation by posterior approach aimed to resect the giant tumor, which was derived from the nerve, and to correct the scoliosis. In surgery, we saw that the mass originated from the L_1_ nerve root and extended into the intervertebral space between L_1_ and L_2_. The posterior and partial paravertebral elements of the lump were removed following the reclamation of a vertebral column using a screw-rod system as an internal fixation by a posterior approach. The mass was 11 cm × 7 cm × 4 cm and well encapsulated, luidity section, and was solid with a tough texture (Fig. 2a). Postoperative pathologic examination confirmed it was a ganglioneuroma (Fig. 2c–f). The second stage operation by the thoracoabdominal anterior-lateral approach was performed 2 months later to eradicate the thoracolumbar paravertebral giant ganglioneuroma. An intraoperative exploration found that it was a retroperitoneal mass, derived from the intervertebral foramen from L_1_ to L_2_, extending T_10_ to L_4_, measuring 13 cm × 8 cm × 6 cm, identical in character to the first mass, the giant tumor (Fig. 2b). The postoperative pathologic examination also presented identically.

**Fig. 3:**
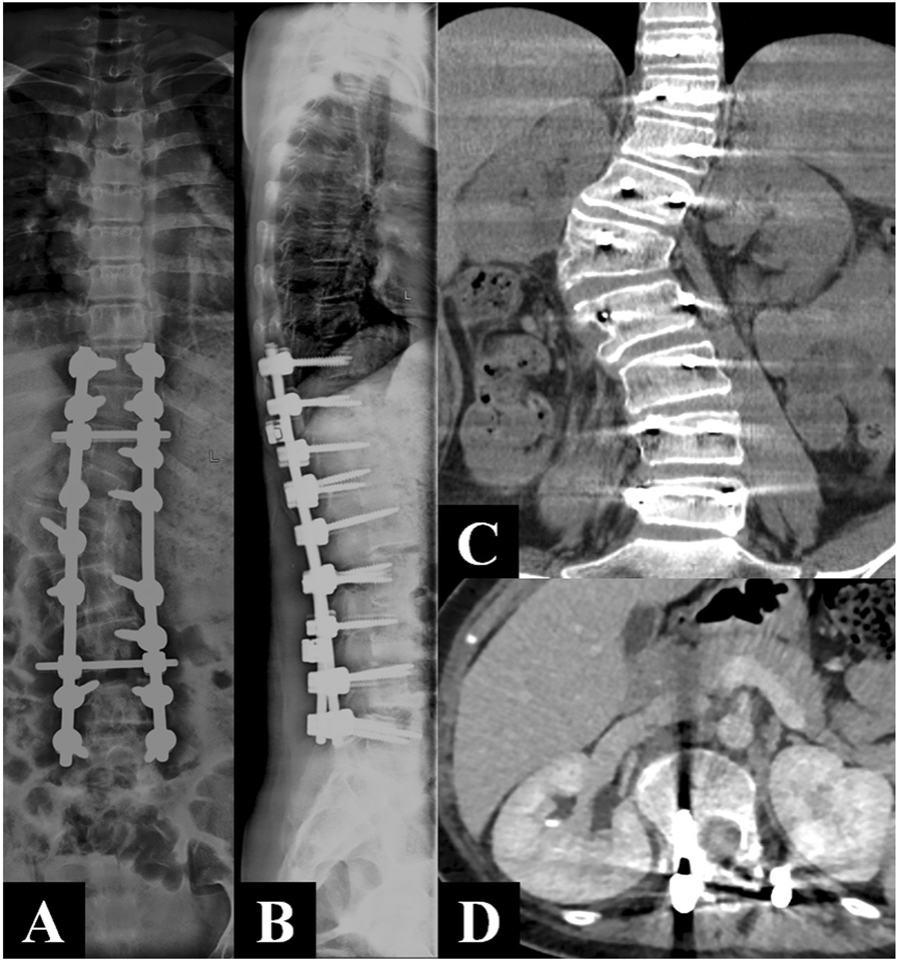
**a**, **b** The instrument which is used for internal fixation formed an image in the vertebral body and paravertebral region. The lower segment of the thoracic and lumbar spine existed a right tumefied thoracolumbar curve surrounding the first lumbar body with a Cobb angle of 15.3° and Ferguson angle of 44.3°. **c**, **d** CT coronary multi-plane reorganization and axial enhanced scan showed the right paravertebral mass had disappeared, and the kidney was lower compared with that before operation. The right kidney vessels are shown clearly

In postoperative plain X-radiographs, the instrument, used for internal fixation, formed an image in the vertebral body and paravertebral region. The lower thoracic and lumbar vertebra surrounding the L_1_ vertebral body maintained a right direction thoracolumbar scoliosis. We measured a Cobb angle of 15.3° and a Ferguson angle of 44.3° (Fig. 3a, b). A further CT revealed that, compared to preoperation, the right direction paravertebral soft tissue clump had disappeared, the right kidney had descended, and the right renal vessels imaging was clearer (Fig. 3c, d). After the staging operation, the patient’s claudication has been disappeared, the patient was 5 cm taller, and her general condition recuperated well.

### Literature review

To review the cases of paravertebral ganglioneuroma and scoliosis, we used “scoliosis” and “ganglioneuroma” as keywords to search Medline for publications from the preceding 40 years. Using the strategy, we found 16 reported cases in 13 papers. For details regarding age, position, clinical characteristics, and follow-up, see Table 1.

**Table 1:** Literature review for the paravertebral ganglioneuroma and scoliosis

References	Sex	Age (year)		Site	Curve pattern	Clinical symptoms		Surgery	Follow-up
		Onset	Diagnosis			First evaluation	During follow-up		
Bauer et al. [16]	F	<12	16	L_1–4_	Right	Backpain	Paraparesis	TR	2 yrs, NR
Rigault et al. [17]									
Case 1	NM	<12	12	NM	Right	No.	No.	TR	11 yrs, NR
Case 2	NM	7	12	NM	Left	No.	No.	TR	2 yrs, NR
Case 3	NM	<5	5	NM	Right	Paraparesis	NM	TR	NM, NR
Sampson et al. [18]	F	10	12	T_4–7_	Right	No.	No.	NM	NM, NM
Cote et al. [19]	F	12	13.8	T_5–8_	Right	No.	Backpain	TR	2 yrs, NR
Lin et al. [20]	F	<9	9	T_12_-L_3_	Right	No.	No.	TR	NM, NM
Choi et al. [4]	F	5	7	T_2_-L_1_	Right	Weakness of both legs	NM	PR	NM, NM
Joachim et al. [21]	M	<13	13	T_6–10_	Right	Backpain	NM	NM	NM, NM
Velyvis et al. [11]	F	<15	15	T_2–7_	Right	Backpain	No.	TR	6 yrs, NR
Lai et al. [2]	F	10	12	T_8–11_	Right	No.	No.	TR	2 yrs, NR
Spiegel et al. [22]	F	<14	14	T_5–7_	Right	No.	No.	TR	2 yrs, NR
Qiu et al. [23]									
Case 1	M	<9	9	T_9_-L_1_	Left	No.	No.	TR	1 yrs, NR
Case 2	F	9	14	T_3–12_	Right	Backpain	No.	TR	2.5 yrs, NR
Kara et al. [24]	M	2	28	T_4–11_	Right	NM	Dyspnea and vomiting	TR	26 yrs, R
D'Eufemia et al. [25]	F	9	11	T_4–11_	Right	No.	No.	PR	2 yrs, NM
Current case	F	3	12	T_10_-L_4_	Right	No.	No.	TR	18 mths, NR

*M* male, *F* female, *mths* months, *yrs* years, *TR* total resection, *PR* partial resection, *NR* no recurrence, *R* recurrence, *NM* not mentioned

### Discussion

Neurogenic tumors may be broadly classified as arising from nerve cells or nerve sheaths. The former group includes ganglioneuroma, ganglioglioma, ganglioneuroblastoma, and neuroblastoma, and the latter includes neurilemmoma, neurofibroma, and malignant schwannoma [5]. In 1941, Eden [6] classified dumbbell-shaped tumors into four categories according to the anatomical relationship, that is, the spinal cord and vertebrae: intradural and extradural; intradural, extradural, and paravertebral; extradural and paravertebral; and foraminal and paravertebral. In this case, the tumor, which was located in the intraspinal region, passes through the intervertebral foramen to form a paravertebral mass resembling a dumbbell. It is extradural and paravertebral according to the Eden categorization. The most common dumbbell-shaped tumor is the Schwann cell tumor, whereas ganglioneuroma is relatively rare [7].

Osteoidosteoma is one of the most common types of scoliosis deformities caused by tumors [8]. The scoliosis caused by osteoidosteoma is mainly connected with pain and paravertebral myositis [9, 10]. But patients with paravertebral ganglioneuroma and scoliosis feel no pain and experience no paravertebral myositis. There are three types of paravertebral ganglioneuroma and scoliosis: (1) the tumor grows expansively, leading to damages in the side and front vertebrae and eventually to scoliosis; (2) scoliosis is mechanically stimulated, induced the tumor; (3) paravertebral ganglioneuroma and scoliosis occur simultaneously. It was reported 60~80 % of dumbbell tumors can cause nerve root compression symptoms, while 20~40 % of the patients had no nerve compression symptoms [11]. Combined with the patient’s medical history and related literatures, in this case, we consider that scoliosis was caused by ganglioneuroma. One possible reason is that the tumor stimulated the affected side vertebral epiphyseal plate, leading to its overgrowth [4]. Another possible reason is that the tumor involved paravertebral muscle of convex side, causing the convex side muscle atrophy [2].

The typical manifestation of ganglioneuroma is low density and punctate calcification on plain CT, showing a high T2 signal on MRI and a gradual increase in enhancement on dynamic images; it presents as non-enhancement or mild enhancement in the arterial phase of CTs or MRIs and progressive mild enhancement in the delayed phase. If ganglioneuroma shows an atypical manifestation on a CT and an MRI, we consider the tumor to contain a malignant component [12]. In recent years, 3D printing technology has proven helpful not only to make an operation plan and simulate the operation in the preoperative stage but also for patients to learn more about their conditions and facilitate communication with doctors [13, 14].

The ganglioneuroma shows complete capsular and basal growth by expansive patterns. The most effective therapy is a surgical removal operation as soon as possible, which can reduce the risk of malignant transformation, paraplegia, and other abnormalities. Moreover, surgery has a good prognosis. If the tumor does not affect the vital organs, it should be removed completely; however, if complete resection may cause serious complications, then a partial resection should be performed [15]. In this case, the preoperative Cobb angle was 33.7°, but due to the combined thoracolumbar paravertebral giant ganglioneuroma, a complete resection required a laminectomy. To maintain the spinal stability, in this case, the patient also required a scoliosis deformity correction and an internal fixation. The postoperative Cobb angle is 15.3°, and it was corrected well. Thoracolumbar paravertebral giant ganglioneuroma and scoliosis make a single surgical approach difficult to correct a spine deformity and resect the tumor completely. Therefore, the operation is divided into two stages. The interval time between two operations should be 1 to 7 weeks [2, 4, 11, 16–25] according to literature reports. However, as for us, the interval time should be considered from patients’ recovery condition after the first operation. If the patient recovers well and shows no signs of surgical contraindication, then the second operation can be performed. A piecemeal resection of the tumor can reduce the injury of paravertebral nerve and muscle. Adopting different surgical approaches can avoid injury of important blood vessels, nerves, and organs, so that the tumor is fully exposed. On the other hand, adopting different surgical approaches is good for operation and shortening the operation time; in addition, patients will better be able to tolerate the operation, while doctors can maintain their physical stamina. And at the same time, it increases the safety during the operation. Paravertebral giant ganglioneuroma and scoliosis usually affect the lung, kidney, intestine, large blood vessels, and other important organs. The postoperative complications include cerebrospinal fluid leakage, paraparesis, intestinal obstruction, perioperative bleeding, and pneumothorax, among other complications [26]. Consequently, multidisciplinary consultation preoperation and intraoperative multidisciplinary joint surgery can lower the risks of developing complications.

Ganglioneuroma has low recurrence rate, a good prognosis, and does not require chemotherapy, radiation therapy, or other treatments following complete resection [27, 28]. The patients should accept ultrasonography, DR, and CT checks regularly for a more thorough evaluation of partial recurrence after the surgery. The patient has been followed up for 18 months and shows a good scoliosis correction, a good internal fixation, and no tumor recurrence. At this point, the patient is still under follow-up.

## Conclusions

Thoracolumbar paravertebral giant ganglioneuroma and scoliosis is quite uncommon. Through clinical manifestations and auxiliary examination, we can make a preliminary diagnosis, but we nevertheless require pathological diagnosis results for confirmation. The treatment of a ganglioneuroma is divided into a two-stage surgical operation: first, the correction of the deformity and then the resection of the tumor. Long-term, postoperative follow-up series indicate a low incidence of recurrence and a good survival rate if the tumor is completely resected.

### Consent

The patient and the families were informed that related data and attached images in the case would be submitted for publication. Since they had signed the Informed Consent Form, its copy can be provided to the journal.

## Abbreviation

CNS: central nervous system
F: female
M: male
mths: months
NM: not mentioned
NR: no recurrence
PR: partial resection
R: recurrence
TR: total resection
yrs: years
